# The Cytomegalovirus-Specific IL-21 ELISpot Correlates with Allograft Function of Kidney Transplant Recipients

**DOI:** 10.3390/ijms19123945

**Published:** 2018-12-08

**Authors:** Monika Lindemann, Johannes Korth, Ming Sun, Shilei Xu, Christoph Struve, Kai Werner, Theresa Dornieden, Peter A. Horn, Oliver Witzke, Benjamin Wilde

**Affiliations:** 1 Institute for Transfusion Medicine, University Hospital, Virchowstraße 179, 45147 Essen, Germany; theresa.dornieden@outlook.de (T.D.); peter.horn@uk-essen.de (P.A.H.); 2 Department of Nephrology, University Hospital Essen, Hufelandstraße 55, 45147 Essen, Germany; johannes.korth@uk-essen.de (J.K.); bluesilenter@hotmail.com (M.S.); xslzssy@163.com (S.X.); christoph.struve@uk-essen.de (C.S.); KaiWerner1987@web.de (K.W.); oliver.witzke@uk-essen.de (O.W.); benjamin.wilde@uk-essen.de (B.W.); 3 Department of Infectious Diseases, University Hospital Essen, Hufelandstraße 55, 45147 Essen, Germany

**Keywords:** human cytomegalovirus, reactivation, ELISpot, interferon-γ, interleukin-21, interleukin-17A, kidney function, sex-related difference

## Abstract

In kidney transplant recipients, the cytomegalovirus (CMV) is frequently causing infection/reactivation and can trigger allograft rejection. To assess the risk of reactivation, the cellular immune response against CMV is increasingly assessed by cellular in vitro methods, such as the interferon (IFN)-γ ELISpot. In the current study we compared the IFN-γ ELISpot with our newly established CMV-specific ELISpot assays determining IL-17A, IL-21, IL-22, granzyme B, and perforin and correlated the results with flow cytometric data and clinical parameters. In 77 kidney transplant recipients, the highest frequency was observed for CMV pp65-specific cells secreting IFN-γ, followed by cells secreting IL-21 (62.9 and 23.2 Δ spot forming cells/10^5^ cells). We observed a positive correlation between the percentage of CMV-specific CD3+ CD4+ CD154+ cells and results of the CMV-specific IL-21 ELISpot (*p* = 0.002). Results of the CMV pp65-specific IL-21 ELISpot correlated negatively with kidney function (estimated glomerular filtration rate, *p* = 0.006) and were significantly higher in women (*p* = 0.005). IL-21, a cytokine involved in aging that is secreted by activated CD4+ T cells, may also impact on allograft function. Thus, the CMV-specific IL-21 ELISpot could become a new tool to assess if CMV seropositivity represents a hazard for the graft.

## 1. Introduction

Following kidney transplantation, infection and allograft rejection are the two major obstacles. Herpesviruses, especially cytomegalovirus (CMV), frequently cause infection or reactivation; leading e.g., to fever, interstitial pneumonitis, hepatitis, colitis, and encephalitis [1] or triggering allograft rejection [2]. Thus, it is essential to closely monitor CMV immunity after transplantation. Apart from determining CMV viral load, the cellular immune response against CMV is increasingly assessed by cellular in vitro methods, such as the ELISpot [3]. This highly sensitive assay can detect cytokine secretion on a single cell level [4] and it is feasible to detect antiviral immunity also in transplant recipients [5]. The quantification of cellular CMV immunity may help to stratify the risk of CMV infection or reactivation, and thereby guide pre-emptive and prophylactic antiviral treatment [6,7].

It is well established that interferon (IFN)-γ is crucial in antiviral control and all cellular assays that are CE-marked for in vitro diagnostics determine CMV-specific IFN-γ production [7,8,9,10,11]. Data on other cytokines, however, are scarce. Interleukin (IL)-17 and IL-21, cytokines regulating T-cell responses, may play a role in CMV-specific immunity. Previous studies showed that IL-17 mRNA levels (in plasma) were significantly increased in CMV positive vs. negative liver transplant recipients [12]. Furthermore, in aged patients chronic CMV infection led to increased IL-21 secretion [13]. IL-22 and effector molecules of cytotoxic T cells are additional candidates that may be involved in CMV-specific cellular immunity. IL-22 down-regulates IFN-γ [14] and could, therefore, counter-regulate CMV-specific antiviral control. Granzyme B and perforin were found to be significantly elevated in bronchoalveolar lavage of lung transplant recipients with vs. without CMV infection [15]. So far, a comparative analysis of the above-mentioned set of cytokines that are (potentially) involved in CMV infection/reactivation or disease is lacking.

The aim of the current study was (I) to compare the frequency of CMV-specific cells producing IFN-γ, IL-17A, IL-21, IL-22, granzyme B, and perforin, (II) to examine whether there was any correlation between the various cytokines, (III) to correlate surface markers of T cells with the frequency of cytokine producing cells, and (IV) to correlate clinical parameters with ELISpot results.

## 2. Results

### 2.1. Frequency of CMV-Specific Cells

**Figure 1:**
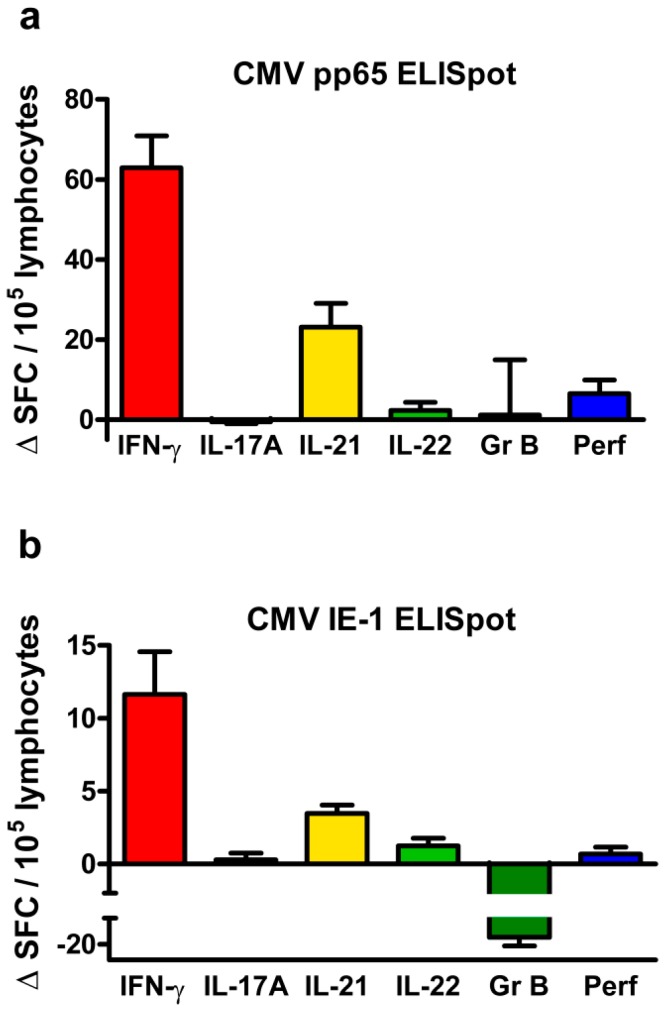
Frequency of cytomegalovirus (CMV)-specific cells in CMV immunoglobulin (Ig)G positive kidney transplant recipients. (**a**) shows ELISpot results as mean and standard error of the mean (SEM) after stimulation with CMV phosphoprotein 65 (pp65) and (**b**) after stimulation with CMV immediate early antigen 1 (IE-1). The frequency is given as delta value of spot forming cells (Δ SFC) per 10^5^ lymphocytes, indicating that autologous (negative) controls were subtracted. One data set per patient was included into these analyses and ELISpot results were normalized, as specified in the *Materials and Methods* section. Patient numbers varied because the amount of cells was not always sufficient to perform all ELISpot assays in parallel [interferon (IFN)-γ and interleukin (IL)-21: *n* = 77; IL-17A: *n* = 58; IL-22, granzyme B (GrB) and perforin (Perf): *n* = 42].

The frequency of CMV-specific cells in adult kidney transplant recipients was analyzed by ELISpot assay and given as spot forming cells (SFC) per 10^5^ cells. Negative controls were subtracted from CMV-specific responses (Δ SFC). CMV-specific relating to the ELISpot means specific for the stimulation with two protein antigens, CMV phosphoprotein 65 (pp65) and CMV immediate early antigen 1 (IE-1). In these CMV immunoglobulin (Ig)G positive patients, CMV pp65-specific cells occurred overall at a higher frequency than IE-1-specific cells (Figure 1). Following stimulation with CMV pp65, cells secreting IFN-γ reached the highest frequency [62.9 ± 8.0 Δ SFC/10^5^ cells, data represent mean and standard error of the mean (SEM)], followed by cells secreting IL-21 (23.2 ± 5.9 Δ SFC/10^5^ cells) (Figure 1a). Whereas IFN-γ and IL-21 secretion was determined in 77 transplant recipients, secretion of IL-17A was determined only in 58 and of IL-22, granzyme B, and perforin in 42 recipients, which limits the significance of the analyses on these latter cytokines. The number of cells secreting IL-22 and perforin was very low and the number of those secreting IL-17A and granzyme B showed high variation between patients; resulting overall in Δ SFC values that are close to or even below zero. Following stimulation with CMV IE-1, the cells mainly produced IFN-γ (11.6 ± 3.0 Δ SFC/10^5^ cells) and IL-21 (3.5 ± 0.6 Δ SFC/10^5^ cells) (Figure 1b).

**Figure 2:**
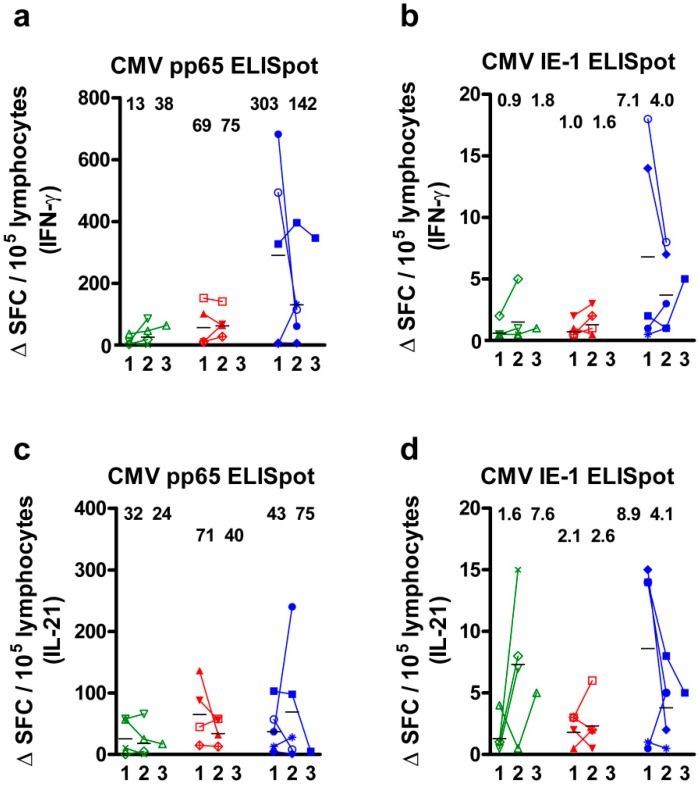
Course of CMV-specific cell numbers in 13 kidney transplant recipients. Patients were grouped into those with a decrease (*n* = 4, marked green), an increase (*n* = 4, red) or constantly undetectable viral load (*n* = 5, blue). Cells were either stimulated by CMV phosphoprotein 65 (pp65) or CMV immediate early antigen 1 (IE-1) and the secretion of IFN-γ (**a**,**b**) or IL-21 (**c**,**d**) was analyzed. The frequency is given as delta value of spot forming cells (Δ SFC) per 10^5^ lymphocytes, indicating that negative (autologous) controls were subtracted. ELISpot responses were determined at up to three consecutive time points (1, 2, and 3). An individual icon was assigned to each patient. Mean values at time point 1 and 2 are indicated as numbers and horizontal lines. Δ SFC below 0 were set as 0.

In 13 CMV IgG positive patients, the course of CMV-specific cell numbers could be analyzed at up to three time points in a longitudinal manner (Figure 2). Patients with a decrease of the CMV viral load (*n* = 4) showed an at least two-fold increase of CMVpp65- and IE-1-specific IFN-γ responses (mean value), whereas patients with an increase of the viral load (*n* = 4) presented a less pronounced increase of IFN-γ ELISpot results (Figure 2a,b). In patients with constantly undetectable viral load (*n* = 5), we observed an overall more than seven-fold higher response than in patients with detectable viral load (pp65: 302.6 Δ SFC/10^5^ cells; IE-1: 7.1 Δ SFC/10^5^ cells), but a decrease over time. CMV-specific IL-21 responses, however, remained overall unchanged (Figure 2c,d). The distance between the first and second measurement was on average four months and between the second and third two months. The analysis of CMV pp65-specific spot numbers at the first and second time point revealed a positive correlation (IFN-γ ELISpot: *r* = 0.54, *p* = 0.06; IL-21: *r* = 0.64, *p* = 0.02). Of note, none of the cytokines was secreted in four CMV IgG negative kidney transplant recipients, indicating CMV-specific cellular responses.

### 2.2. Correlation of Results to Various CMV-Specific ELISpot Assays

**Figure 3:**
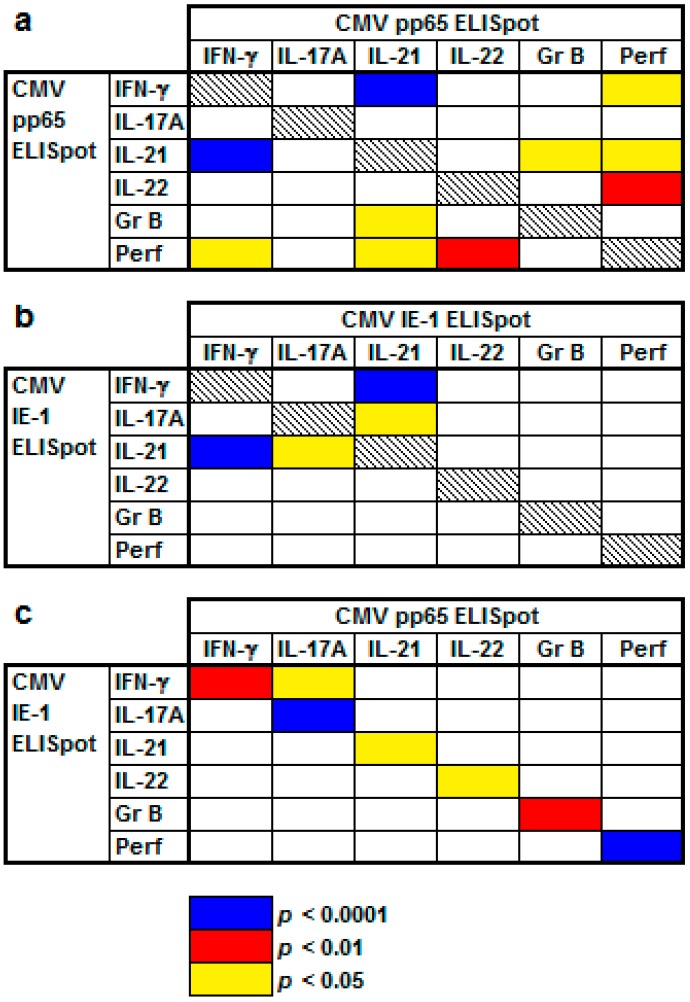
Positive correlation of CMV-specific ELISpot results in kidney transplant recipients. (**a**) indicates results after stimulation with CMV phosphoprotein 65 (pp65), (**b**) after stimulation with CMV immediate early antigen 1 (IE-1) and (**c**) after stimulation with both CMV antigens. This Spearman correlation analysis considers 77 CMV IgG positive kidney transplant recipients and includes one data set per patient, as specified in the *Materials and Methods* section. Further details on this analysis are given in Table S1 (Supplement).

Spearman analysis of CMV IgG positive kidney transplant recipients (*n* = 77) indicated that there were positive correlations between various ELISpot assays (Figure 3). Following stimulation with CMV pp65, correlations were highly significant for IFN-γ and IL-21 (*r* = 0.53, *p* < 0.0001) and IL-22 and perforin (*r* = 0.50, *p* = 0.001) (Figure 3a). Following stimulation with CMV IE-1, correlations were highly significant for IFN-γ and IL-21 (*r* = 0.51, *p* < 0.0001, Figure 3b). In addition, the cytokine responses upon stimulation with the CMV antigens pp65 and IE1 were correlated positively with each other (*p* < 0.05 each, Figure 3c). However, CMV-specific IFN-γ and granzyme B ELISpot correlated negatively (IE-1: *r* = −0.32, *p* = 0.04) and CMV-specific IFN-γ and IL-22 ELISpot tended to correlate negatively (IE-1: *r* = −0.23, *p* = 0.1), as shown in Table S1. The majority of correlations were positive, whereas a few were negative, especially those with granzyme B and IL-22 (Table S1).

### 2.3. Correlation between Results to Flow Cytometry and CMV-Specific ELISpot Assays

**Figure 4:**
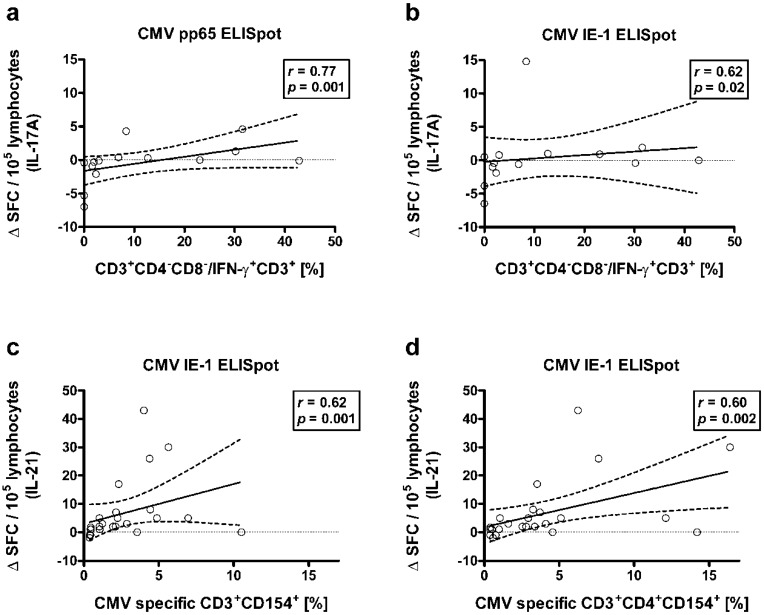
Spearman correlation of flow cytometric and ELISpot data. (**a**,**b**) Correlation between the percentage of CD4 CD8 double negative cells within the CD3+ T cells secreting IFN-γ and the proportion of CMV-specific cells secreting IL-17A in response to CMV pp65 or IE-1, respectively (*n* = 14). (**c**,**d**) Correlation between the percentage of CMV-specific CD3+ CD154+ cells or of CMV-specific CD3+ CD4+ CD154+ cells and the proportion of CMV-specific cells secreting IL-21 in response to CMV IE-1, respectively (*n* = 24). The diagonal continuous line represents the regression line, the dashed lines the 95% confidence interval. The horizontal dotted line is set at zero.

Combining flow cytometric and ELISpot data, we observed that there was a positive correlation between the percentage of CD4 CD8 double negative cells within the CD3+ T cells secreting IFN-γ and the number of CMV-specific cells secreting IL-17A (*n* = 14). The correlation of these double negative T cells with CMV pp65-specific cells was stronger than with CMV IE-1-specific cells (*r* = 0.77, *p* = 0.001 and *r* = 0.62, *p* = 0.02) (Figure 4a,b). Results were similar when considering the absolute numbers of CD4 CD8 double negative cells instead of percentages (*r* = 0.72, *p* = 0.004, and *r* = 0.39, *p* = 0.17, respectively). Presumably, IFN-γ secreting CD4 CD8 double negative cells could also secrete IL-17A or induce IL-17A secretion upon CMV-specific stimulation. Of note, IFN-γ secreting CD4 CD8 double negative cells represented only 4.9% of the CD3+ T cells secreting IFN-γ (median value).

Moreover, in 24 patients various T cell subsets expressing CD154 as activation marker were correlated with ELISpot data. The strongest correlation was found for the percentage of CMV-specific CD3+ CD154+ cells and results of the IL-21 ELISpot (CMV IE-1) (*r* = 0.62, *p* = 0.001) (Figure 4c). Similar results were obtained for the CD4+ subset of these cells (*r* = 0.60, *p* = 0.002) (Figure 4d), whereas the correlation with the CD8+ subset was weaker (*r* = 0.42, *p* = 0.04). Furthermore, the percentage of CMV-specific CD3+ CD4+ CD154+ cells significantly correlated with results to the IFN-γ ELISpot (CMV pp65) (*r* = 0.50, *p* = 0.01). Thus, activated CD4+ T cells may be a source of CMV-specific IL-21 and IFN-γ production.

### 2.4. Correlation of Clinical Parameters and ELISpot Results

The interval between sampling and kidney transplantation, estimated glomerular filtration rate (eGFR) and dosage or administration (yes/no) of immunosuppressive drugs (prednisone, cyclosporine A, tacrolimus, mycophenolate mofetil, mTOR inhibitors) were included as clinical parameters. Furthermore, the presence of detectable CMV viral load, symptomatic CMV infection, CMV syndrome, and invasive CMV disease, respectively, as well as patients’ sex were considered. One data set per patient was included as detailed in the *Materials and Methods* section. These parameters were considered either prior to sampling, at sampling or within three months after sampling as indicated.

**Figure 5:**
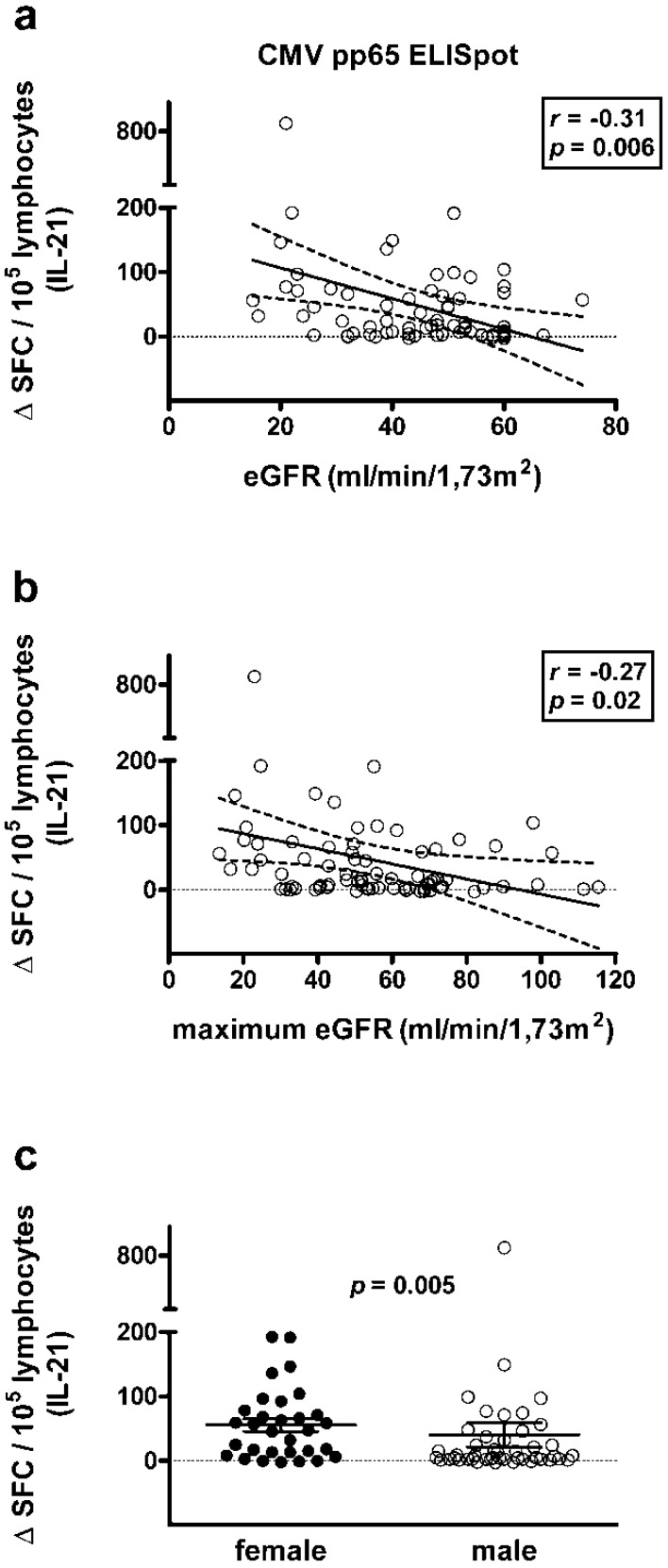
Correlation between kidney function or patient sex and CMV pp65-specific IL-21 ELISpot responses. (**a**,**b**) Correlation between estimated glomerular filtration rate (eGFR) at sampling and the maximum value within three months after sampling and the proportion of CMV pp65-specific cells secreting IL-21, respectively. (**c**) Comparison of results to the CMV pp65-specific IL-21 ELISpot in females (*n* = 31) and males (*n* = 46), as analyzed by the Mann-Whitney *U* test. The diagonal continuous line represents the regression line, the dashed lines the 95% confidence interval (**a**,**b**). The horizontal continuous lines indicate the mean value and standard error of the mean (SEM) (**c**). The horizontal dotted line is set at zero.

Spearman correlation analysis showed that kidney function (eGFR) at sampling and the maximum value within three months after sampling were negatively correlated with results of the CMV pp65-specific IL-21 ELISpot (*r* = −0.31, *p* = 0.006 and *r* = −0.27, *p* = 0.02, respectively), i.e., IL-21 secretion was lower in patients with better kidney function (Figure 5a,b). Remarkably, results in none of the other CMV-specific ELISpot assays correlated with kidney function, e.g., for IFN-γ the correlation coefficient *r* was between 0 and −0.08. Finally, the interval between sampling and kidney transplantation did not show any correlation with the ELISpot results.

The major results on categorial variables are compiled Table 1. Table S2 contains all results that were obtained by the Mann-Whitney *U* test. Results to the IFN-γ ELISpot were almost six-fold lower in patients with vs. without detectable CMV viral load prior to sampling (IE-1: 6.5 vs. 37.2 Δ SFC, *p* = 0.03). Results to the IL-22 ELISpot were almost eight-fold higher in patients with and without detectable symptomatic CMV infection prior to sampling (IE-1: 5.4 vs. 0.7 Δ SFC, *p* = 0.02). Furthermore, patients with detectable CMV viral load prior to sampling had significantly lower kidney function (eGFR) at sampling (*p* = 0.001) and within three months after sampling (*p* = 0.009), respectively. Similar results were obtained for patients with CMV-related diseases. In patients with symptomatic CMV infection and with invasive CMV disease prior to sampling, kidney function was significantly impaired (*p* < 0.05) (Table S2). The dosage of immunosuppressive drugs also correlated with CMV viral load and CMV-related disease. The dosage of prednisone was significantly higher in patients with detectable CMV viral load (*p* = 0.02) or symptomatic CMV infection (*p* = 0.008) within three months after sampling. Patients with detectable viral load prior to sampling received significantly (*p* = 0.04) more mycophenolate mofetil (MMF). Vice versa, patients with detectable CMV viral load prior to sampling had received a lower dose of mTOR inhibitors, which however was not statistically significant (*p* = 0.16). Patients with vs. without cyclosporine A, tacrolimus, mycophenolate mofetil, or mTOR inhibitor treatment, respectively, had overall similar results to the ELISpot and similar kidney function. Finally, females showed significantly higher responses to the IL-21 ELISpot than males (pp65: 55.4 vs. 40.1 Δ SFC, *p* = 0.005) (Figure 5c).

## 3. Discussion

Among the cytokine-producing cells that were investigated in the current study, those producing IFN-γ and IL-21 in response to CMV antigens were dominant. CMV pp65-specific responses reached a mean frequency of 62.9 and 23.2 Δ SFC/10^5^ cells in the IFN-γ and IL-21 ELISpot, respectively. Previously, in kidney transplant recipients [7] and in healthy controls [16] frequencies of CMV-specific, IFN-γ secreting cells were reported at a similar level. However, data on the frequency of CMV-specific, IL-21 secreting cells are currently not available.

Correlation between IFN-γ and IL-21 ELISpot results showed the highest correlation coefficients (*r* = 0.53 for CMV pp65 stimulation). Thus, both cytokines could be functionally related. IL-21 is known as a cytokine regulating T cell responses. It is primarily secreted by CD4+ T cells (in particular follicular T cells and Th17 cells). For Th17 cells, IL-21 is an autocrine regulator sustaining Th17 cell development [17]. IL-21 is, for example, used together with IL-2 and IL-15 to massively expand CMV-specific CD4+ effector T cells [18]. Our flow cytometric data support these implications. Indeed, we found that the number of CMV-specific cells secreting IL-21 in response to CMV IE-1 was positively correlated with the percentage of CMV-specific CD4+ CD154+ T cells. Agrawal et al. previously reported an increased IL-21 secretion by aged CD4+ T cells, which was associated with prolonged STAT-4 activation and CMV seropositivity [13]. In addition, it was suggested that CMV drives or at least exacerbates immunosenescence long-term after infection [19]. Dysfunctional T cells, as defined by loss of cytokine secretion ability and limited proliferation capacity, were observed after CMV infection [20,21,22]. Moreover, after heart transplantation in vitro and in vivo alloreactivity (mixed lymphocyte reaction and rejection) was associated with increased IL-21 mRNA expression, respectively [23]. Data on CMV-specific IL-21 secretion in transplant recipients have not been reported yet. Also, in kidney transplant recipients, IL-21 could be involved in aging and in alloresponses. CMV-specific IL-21 secretion may accelerate the aging of the graft. This hypothesis is in line with results of our correlation analysis. We observed a negative correlation between the CMV pp65-specific IL-21 ELISpot and kidney function (*p* = 0.006). Thus, high spot numbers correlated with impaired kidney function. A large multicenter study reported that in 727 out of 917 (79%) of kidney transplantations, either the donor or recipient was CMV seropositive [24], and thus at risk of CMV infection/reactivation. In these patients, a CMV pp65-specific IL-21 ELISpot could help to stratify the risk of an impairment of kidney function, which would be clinically highly relevant. If confirmed by independent studies, the CMV pp65-specific IL-21 ELISpot may be a novel tool to assess if CMV seropositivity means hazard for the graft. Importantly, in the current cohort CMV-specific IFN-γ secretion did not correlate with kidney function. Thus, IL-21 may be more sensitive for impaired allograft function.

We observed that numbers of CMV-specific IL-17A spots were highly variable and that the mean value was close to zero. To further elucidate this finding, flow cytometric data were correlated with data on the IL-17A ELISpot. The percentage of CD4 CD8 double negative cells within the CD3+ T cells secreting IFN-γ and the number of CMV-specific cells secreting IL-17A were positively correlated (pp65: *r* = 0.77, *p* = 0.001). CD4 CD8 double negative cells within the CD3+ T cells secreting IFN-γ cells appear as a small but interesting fraction, representing 4.9% of the CD3+ T cells secreting IFN-γ. We wanted to find out whether the double negative cells produced IL-17. Unfortunately, staining for IL-17 in the flow cytometric analysis failed. The detection of IL-17A-secreting cells with the ELISpot was also challenging. As compared to the IFN-γ ELISpot, we had to double cell numbers and to triple the incubation period. Thus, due to its low amount, IL-17 might not have been detectable by flow cytometry. These CD4 CD8 double negative T cells may represent a subset of NKT cells [25], which are expected to be stimulated in our CMV ELISpot assay. We use T-activated^®^ proteins (formulated with Lophius Biosciences’ patented T-activation buffer) for the stimulation of peripheral blood nuclear cells (PBMC), which are processed and cross-presented via human leukocyte antigen (HLA) class I and II, thus mimicking a natural infection [10,26]. These proteins stimulate a broad spectrum of clinically-relevant subpopulations of antigen-reactive effector cells, including T cells but also NK and NKT cells. CD4 CD8 double negative NKT cells could be cytotoxic to CMV-infected cells in a dual Killer cell Ig-like receptor (KIR)- and T cell receptor (TCR)-dependent manner [27]. For a subset of NKT cells expressing an invariant TCR-chain rearrangement (type I NKT or invariant NKT) IL-17 secretion has been described, named iNKT17 cells [28,29]. Responses to CMV are highly variable and involve both innate and adaptive immunity, as described recently in detail [30]. For example, inhibitory NK cell KIR genotypes have been described as a predisposing factor during CMV reactivation in kidney transplantation. A subset of transplant recipients with detectable CMV-specific IL-17A responses could possess these iNKT17 cells, responding to CMV. Patients with detectable CMV viral load and with invasive CMV disease after sampling tended (*p* = 0.1) to have lower responses to the CMV IE-1-specific IL-17A ELISpot, indicating that CMV-specific IL-17 secretion may be involved in the control of CMV infection.

According to a previous publication [14], IL-22 counter-regulates IFN-γ secretion. It was, therefore, expected that we observe a negative correlation between results to the CMV-specific IFN-γ and IL-22 ELISpot. Furthermore, it was reported that immunosenescent cells produce less granzyme B in response to influenza vaccination [19]. Thus, it is possible that the CMV-specific cells detected in our ex vivo assays also display immunosenescence, leading to decreased granzyme B secretion. Overall, we observed CMV IE-1-specific granzyme B secretion even below the negative control. In line with this hypothesis, age tended to be negatively correlated with granzyme B production in response to CMV IE-1 stimulation (*r* = −0.20, *p* = 0.2).

In 13 patients, the course of CMV-specific cell numbers was compared with changes in CMV viral load. As expected, those patients who were able to control the virus showed a more pronounced increase of IFN-γ responses. This observation is compatible with the previous finding that kidney transplant recipients who were protected from CMV reactivation had significantly higher CMV-specific ELISpot responses [6,7,10]. Presumably, in patients with constantly undetectable viral load, there was no stimulus for CMV-specific cells to expand, leading to a decrease over time.

Correlation analyses with clinical endpoints of CMV infection indicated that the CMV-specific IFN-γ ELISpot was most relevant. It has been previously described that CMV replication and CMV disease correlate with impaired kidney function, rejection, and graft loss [24,30]. Our study could confirm these findings. Kidney function was significantly lower in patients with detectable CMV viral load and CMV disease. Moreover, patients with detectable CMV viral load and CMV disease received a significantly higher dosage of immunosuppressive drugs, as expected [31]. We especially looked at the influence of mTOR inhibitors on cellular CMV immunity, because two systematic reviews and meta-analyses showed that the incidence of CMV infection/disease was lower among patients receiving immunosuppressive regimens containing mTOR inhibitors [32,33]. Indeed, those patients without detectable CMV viral load had received a higher dose of mTOR inhibitors (*p* = 0.16). Presumably, this result escaped statistical significance, because only twelve patients were treated by mTOR inhibitors.

Moreover, we observed for the first time a difference in CMV pp65-specific IL-21 secretion between women and men. CMV pp65-specific IL-21 ELISpot responses were significantly higher in females (*p* = 0.005). Previously, higher CMV-specific IL-2 secretion has been reported in females [34]. Furthermore, Bernin et al. reported that the NKT cell ligand α-Galactosylceramide induced higher production of intracellular IFN-γ, IL-4, IL-17, and TNF by CD4+ and CD4 CD8 double negative NKT cells in women [35]. Di Benedetto et al. suggested that there is a sex-related difference in the immunological impact of CMV and reported that in females and males CMV-infection had a different effect on T-cell subpopulations [36]. Interestingly, it was also postulated that the mechanisms by which CMV predisposes to coronary artery disease may be different in men and women [37]. Thus, the sex-related difference in CMV pp65-specific cytokine secretion was not unexpected and should be further examined.

## 4. Materials and Methods

### 4.1. Patients

In total, 77 CMV IgG positive kidney transplant recipients (31 female, 46 male) with a median age of 58 years (range 19–80) were tested for their cellular immunity against CMV by ELISpot assay. Seventy-five percent of the patients were older than 50 years. The median interval to kidney transplantation was 21 months (range 2–337). At sampling for the ELISpot, the median eGFR was 48 mL/min/1.73 m^2^ (as determined by MDRD formula, 15–74). Six patients were treated with cyclosporine A (whole blood trough level 70–150 ng/mL) and 65 with tacrolimus (3–12 ng/mL). 62 patients received mycophenolate mofetil (0.1–24 µg/mL of the catabolic product mycophenolic acid) and twelve mTOR inhibitors [4–8 ng/mL everolimus (*n* = 11) or 6 ng/mL sirolimus (*n* = 1)]. Thus, the majority was treated with tacrolimus and MMF. Additionally, all but two patients received prednisone (median dose 5 mg/day, range 2.5–15). Sixty-four patients were tested once, eleven twice and two trice. If patients were tested more than once, only results that were obtained parallel to flow cytometric data were considered if not otherwise specified. In patients without flow cytometric data the most recent dataset was chosen. Thus, one data set per patient was considered for the assessment of the frequency of CMV-specific cells, for the correlation analysis of various ELISpot assays, and for the correlation with flow cytometric or clinical data. Four CMV IgG negative kidney transplant recipients served as negative controls. Fourteen CMV IgG positive patients were also tested by flow cytometry for IFN-γ secreting T cells and 24 patients for various activated T cell subsets. This study was approved by the institutional review board of the University Hospital Essen (16-7229-BO) and informed consent was obtained from all participants. It was carried out in accordance with the Declarations of Helsinki and Istanbul.

### 4.2. ELISpot Assay

Thirty ml of heparinized blood were collected and peripheral blood mononuclear cells were separated by Ficoll gradient centrifugation. Numbers of PBMC and lymphocytes were determined by an automated hematology analyzer (KX-21N, Sysmex, Norderstett, Germany). Duplicate cultures of freshly isolated PBMC were grown without and with CMV pp65 and CMV IE-1 T-activated^®^ proteins (Lophius Biosciences, Regensburg, Germany). The production of IFN-γ, IL-17A, IL-21, IL-22, granzyme B, and perforin was determined using pre-coated ELISpot plates and a standardized detection system (T-Track^®^ ELISpot kit, Lophius Biosciences). PBMC were incubated without and with CMV proteins in 200 µL AIMV medium (Gibco, Grand Island, NY, USA) at 37 °C. The production of all cytokines except for IL-17A was determined after 19 h of cell culture. To measure IL-17A, cells were pre-incubated overnight in U bottom plates (BD Falcon, Nijmegen, Netherlands). Thereafter, they were incubated for further 48 h in the ELISpot plates. Colorimetric detection of cytokine secreting cells was performed according to the manufacturer´s instructions. ELISpots for IFN-γ and IL-21 were performed with PBMC containing 200,000 lymphocytes, for IL-22, granzyme B and perforin with 100,000, and for IL-17A with 400,000 lymphocytes. These cell numbers could be defined as optimal after titration experiments. Spot numbers were analyzed by an ELISpot reader (AID Fluorospot, Autoimmun Diagnostika GmbH, Strassberg, Germany). Spot counts from the negative (unstimulated) control were subtracted from those of the respective pp65- and IE-1-stimulated conditions and ELISpot results were expressed as Δ SFC per 10^5^ lymphocytes.

### 4.3. Flow Cytometry

Peripheral blood was collected in heparin coated tubes. Whole blood was either stimulated with CMV cell lysate (10 µg/mL) or control lysate (10 µg/mL, HEL-299) (both Lophius Biosciences) for six hours in the presence of anti-human CD28/CD49d (1 µg/mL, clones L293 and L25, BD Biosciences, Heidelberg, Germany). If cytokine production of antigen specific T-cells was determined, Brefeldin A (Sigma Aldrich, Munich, Germany) was added after the first two hours of incubation. Following incubation, whole blood was lysed (FACS lysing solution, BD Biosciences) and permeabilized (FACS Permeabilizing Solution 2, BD Biosciences). Cells were then stained for lineage markers (anti-human CD3, Pacific Blue, clone UCHT1, and anti-human CD4 APC, clone 13B8.2, both Beckman Coulter Krefeld, Germany; anti-CD8 APC-H7, clone SK1, BD Biosciences) and IFN-γ (anti-IFN-γ, FITC, clone 45.15, Beckman Coulter). If CD154 expression was determined on antigen-specific T-cells, whole blood was washed after six hours of incubation and then stained for the above-mentioned lineage-markers, as well as for CD154 (anti-human CD154 FITC, clone 24–31, Biolegend, San Diego, CA, USA). Whole blood was lysed (VersaLyse, Beckman Coulter) and washed twice. Samples were then measured with a flow cytometer (Navios, Beckman Coulter). Data was analyzed using Kaluza Software Version 1.5a (Beckman Coulter).

### 4.4. Statistical Analysis

To evaluate ELISpot results mean values of duplicate cell cultures were considered. CMV-specific spots were determined as CMV-stimulated minus non-stimulated values (delta value, Δ). Subsequently, the number of spot-forming cells (SFC) per 100,000 lymphocytes was calculated. Data were analyzed using GraphPad Prism version 5.03 for Windows (GraphPad Prism Software, La Jolla, CA, USA) or IBM SPSS Statistics version 22 (Armonk, NY, USA). Correlation analyses were performed by Spearman test (two-tailed). Results in patients with vs. without certain clinical parameters were compared by the Mann-Whitney *U* test. If not otherwise stated, mean values are indicated. Results were considered significant at *p* < 0.05.

## 5. Conclusions

This study on cellular CMV-specific immunity in kidney transplant recipients is the first showing that IL-21 is secreted at a considerable number after stimulation with CMV pp65, resulting in an average of 23.2 Δ SFC/10^5^ lymphocytes. We observed a highly significant, negative correlation of kidney function with CMV pp65-specific IL-21 spot numbers. As IL-21 is a regulator of T cells that is involved in aging and alloresponses, it may impact on allograft function. Thus, if confirmed, the CMV pp65-specific IL-21 ELISpot could become a new tool to assess whether CMV seropositivity represents a hazard for the graft. Finally, responses to the CMV pp65-specific IL-21 ELISpot were significantly higher in females, which may negatively influence the outcome in female kidney transplant recipients.

## Figures and Tables

**Table 1:** Major results on categorial variables as obtained in 77 kidney transplant recipients.

Variable 1	Variable 2	*p* Value
Detectable CMV viral load ^0^	IFN-γ IE-1 ELISpot	0.03
Symptomatic CMV infection ^0^	IL-22 IE-1 ELISpot	0.02
Detectable CMV viral load ^0^	eGFR ^1^	0.001
Detectable CMV viral load ^0^	Max. eGFR ^2^	0.009
Detectable CMV viral load ^2^	Dosage of prednisone ^1^	0.02
Symptomatic CMV infection ^2^	Dosage of prednisone ^1^	0.008
Patient sex	IL-21 pp65 ELISpot	0.005

^0^ prior to sampling (for the ELISpot); ^1^ at sampling; ^2^ within 3 months after sampling; eGFR = estimated glomerular filtration rate.

## Supplementary Materials

Supplementary Materials can be found at http://www.mdpi.com/1422-0067/19/12/3945/s1.

## Abbreviations

CMV	cytomegalovirus
eGFR	estimated glomerular filtration rate
HLA	human leukocyte antigen
IE-1	immediate early antigen 1
IFN	interferon
IL	interleukin
Ig	immunoglobulin
MMF	mycophenolate mofetil
mTOR	mechanistic (mammalian) Target of Rapamycin
pp65	phosphoprotein 65
SEM	standard error of the mean
SFC	spot forming cells
