# Supplementary material for: The Cytomegalovirus-Specific IL-21 ELISpot Correlates with Allograft Function of Kidney Transplant Recipients
Source: Int J Mol Sci. 2018 Dec 8;19(12):3945. doi: 10.3390/ijms19123945 (PMC6320857; doi:10.3390/ijms19123945)
Supplement: Supplementary file 1 [file ijms-19-03945-s001.pdf]

Table S1 Spearman correlation analysis of CMV ELISpot results

		IFN-γ IE1	IFN-γ pp65	IL-17 IE1	IL-17 pp65	IL-21 IE1	IL-21 pp65	IL-22 IE1	IL-22 pp65	GrB IE1	GrB pp65	Perf IE1	Perf pp65
IFN-γ IE1	*r*	1.00	.32**	.05	.24*	.51**	.15	.02	.25	.06	-.05	.09	.07
	p		.005	.68	.03	.0000	.18	.90	.11	.69	.78	.57	.67
	n	77	77	77	77	77	77	41	41	41	41	41	41
IFN-γ pp65	*r*	.32**	1.00	.12	.13	.14	.53**	-.23	.19	-.32	.08	.20	.32*
	p	.005		.31	.26	.24	.0000	.14	.22	.04	.64	.20	.04
	n	77	77	77	77	77	77	41	41	41	41	41	41
IL-17 IE1	*r*	0.05	0.12	1.00	.47**	.27*	0.18	0.21	0.28	0.12	0.01	0.13	0.14
	p	.68	.31		.0000	.02	.12	.19	.08	.47	.96	.41	.37
	n	77	77	77	77	77	77	41	41	41	41	41	41
IL-17 pp65	*r*	.24*	0.13	.47**	1.00	0.15	0.14	-0.08	0.26	0.13	0.22	0.21	0.25
	p	.03	.26	.0000		.19	.21	.62	.10	.40	.17	.18	.11
	n	77	77	77	77	77	77	41	41	41	41	41	41
IL-21 IE1	*r*	.51**	0.14	.27*	0.15	1.00	.26*	-0.20	-0.05	0.00	-0.07	0.06	0.10
	p	.0000	.24	.02	.19		.02	.22	.75	.99	.65	.72	.54
	n	77	77	77	77	77	77	41	41	41	41	41	41
IL-21 pp65	*r*	0.15	.53**	0.18	0.14	.26*	1.00	-0.20	0.17	-0.01	.39*	0.30	.33*
	p	.18	.0000	.12	.21	.02		.22	.29	.93	.01	.06	.03
	n	77	77	77	77	77	77	41	41	41	41	41	41
IL-22 IE1	*r*	0.02	-0.23	0.21	-0.08	-0.20	-0.20	1.00	.34*	0.01	-0.06	-0.02	0.00
	p	.90	.14	.19	.62	.22	.22		.03	.96	.69	.89	.98
	n	41	41	41	41	41	41	41	41	41	41	41	41
IL-22 pp65	*r*	0.25	0.19	0.28	0.26	-0.05	0.17	.34*	1.00	0.06	0.22	0.26	.50**
	p	.11	.22	.08	.10	.75	.29	.03		.69	.17	.10	.001
	n	41	41	41	41	41	41	41	41	41	41	41	41
GrB IE1	*r*	0.06	-0.32	0.12	0.13	0.00	-0.01	0.01	0.06	1.00	.51**	0.13	0.03
	p	.69	.04	.47	.40	.99	.93	.96	.69		.001	.41	.84
	n	41	41	41	41	41	41	41	41	41	41	41	41
GrB pp65	*r*	-0.05	0.08	0.01	0.22	-0.07	.39*	-0.06	0.22	.51**	1.00	0.17	0.28
	p	.78	.64	.96	.17	.65	.01	.69	.17	.001		.29	.08
	n	41	41	41	41	41	41	41	41	41	41	41	41
Perf IE1	*r*	0.09	0.20	0.13	0.21	0.06	0.30	-0.02	0.26	0.13	0.17	1.00	.58**
	p	.57	.20	.41	.18	.72	.06	.89	.10	.41	.29		.000
	n	41	41	41	41	41	41	41	41	41	41	41	41
Perf pp65	*r*	0.07	.32*	0.14	0.25	0.10	.33*	0.00	.50**	0.03	0.28	.58**	1.00
	p	.67	.04	.37	.11	.54	.03	.98	.001	.84	.08	.0001	
	n	41	41	41	41	41	41	41	41	41	41	41	41

Correlation of CMV-specific ELISpot results in 77 CMV IgG positive kidney transplant recipients.
Negative correlations are marked in light blue, positive correlations reaching statistical significance in yellow (*$p < 0.05$, **$p < 0.01$).
The Spearman correlation analysis was performed 2-tailed. Patient numbers vary because the amount of cells was not always sufficient to perform all ELISpot assays in parallel.
GrB = Granzyme B; Perf =Perforin; IE-1 = CMV Immeadiate Early Antigen 1; pp65 = CMV Phosphoprotein 65.

Table S2

Analysis of categorial variables (Mann Whitney *U* test)

Categorial variable	*p values*																		
	ELISpot results												Kidney function		Dosage at sampling				
	IFN-γ IE1	IFN-γ pp65	IL-17 IE1	IL-17 pp65	IL-21 IE1	IL-21 pp65	IL-22 IE1	IL-22 pp65	GrB IE1	GrB pp65	Perf IE1	Perf pp65	eGFR[1]	max. eGFR[2]	Pred-nison	CsA	FK	MMF	mTOR
**Prior to sampling**																			
Detectable CMV viral load	.03	.08	.99	.82	.81	.29	.30	.26	.92	.40	.79	.96	.001	.009	.12	.27	.27	.04	.16
Symptomatic CMV infection	.13	.36	.44	.96	.20	.14	.02	.79	.43	.03	.78	.61	.03	.03	.62	.06	.58	.52	.57
CMV syndrome	.31	.47	.90	.73	.42	.19	.23	.22	.48	.05	.51	.07	.10	.06	.78	.005	.23	.45	.99
Invasive CMV disease	.66	.65	.55	.95	.06	.15	.18	.54	.76	.15	.11	.74	.03	.03	.24	.81	.63	.53	.23
**Within three months after sampling**																			
Detectable CMV viral load	.47	.18	.08	.97	.83	.34	.62	.19	.47	.66	.42	.91	.001	.008	.02	.64	.91	.10	.30
Symptomatic CMV infection	.85	.06	.42	.71	.26	.23	.67	.39	.55	.39	.84	1.00	.35	.49	.008	.69	.47	.41	.27
CMV syndrome	.85	.06	.42	.71	.26	.23	.67	.39	.55	.39	.84	1.00	.35	.49	.008	.69	.47	.41	.27
Invasive CMV disease	.43	.07	.11	.34	.39	.65	.31	.33	.35	.31	.58	.83	.55	.61	.61	.10	.06	.19	.54
**Patient sex**	.36	.45	.85	.25	.20	.005	.40	.13	.28	.94	.04	.54	.93	.33	.02	.19	.09	.04	.95
**Immunosuppressvie drugs (with vs. without)[3]**																			
CsA, *n* = 6	.97	.14	.68	.34	.93	.41	.34	.76	.92	.74	.71	.98	.60	.55					
Fk, *n* = 65	.27	.90	.61	.54	.11	.12	.63	.96	.77	.59	.74	.78	.42	.29					
MMF, *n* = 62	.19	.95	.97	.55	.69	.33	.15	.06	.67	.64	.30	.53	.53	.40					
mTOR, *n* = 12	.11	.10	.58	.60	.74	.21	.03	.25	.84	.84	.17	.96	.74	.99					

Correlation of clinical parameters and ELISpot results in 77 CMV IgG positive kidney transplant recipients.
Significant results are marked in plain yellow, those close to significance in light yellow.

[1] estimated glomerular filtration rate at sampling (for the ELISpot);

[2] maximum estimated glomerular filtration rate within 3 months after sampling;

[3] numbers indicate patients treated with the respective drug;

CsA = cyclosporin A; Fk = tacrolimus; MMF = mycophenolate mofetil; mTOR = mechanistic (mammalian) Target of Rapamycin.

Table S2 continued

Mean values if results were significantly different or differences were close to significance

		Spots increment per 100.000 cells												ml/min/1,73m²		mg/d	ng/ml	ng/ml	µg/ml	ng/ml
		IFN-γ IE1	IFN-γ pp65	IL-17 IE1	IL-17 pp65	IL-21 IE1	IL-21 pp65	IL-22 IE1	IL-22 pp65	GrB IE1	GrB pp65	Perf IE1	Perf pp65	eGFR[1]	max. eGFR[2]	Pred-nison	CsA	FK	MMF	mTOR
**Prior to sampling**																				
Detectable CMV viral load	+ (*n* = 35)	6.5	91.8											39.6	47.3	5.9			4.1	.6
	Ø (*n* = 42)	37.2	154.3											50.1	60.9	5.4			2.3	1.2
Symptomatic CMV infection	+ (*n* = 11)	1.6					55.2	5.4			10.0			37.1	41.1		21.1			
	Ø (*n* = 66)	26.8					44.8	.7			.1			46.7	57.0		7.5			
CMV syndrome	+ (*n* = 7)										15.7		5.7	36.7	39.1		33.14			
	Ø (*n* = 70)										.2		6.8	46.2	56.3		7.09			
Invasive CMV disease	+ (*n* = 7)					11.9	49.6				5.3	-1.0		34.3	38.1					
	Ø (*n* = 70)					6.8	45.9				1.0	.9		46.5	56.4					
**Within three months after sampling**																				
Detectable CMV viral load	+ (*n* = 22)		74.4	-3.6										38.5	44.1	6.6			4.6	
	Ø (*n* = 55)		146.5	3.2										48.1	58.9	5.2			2.6	
Symptomatic CMV infection	+ (*n* = 6)		41.3													8.3				
	Ø (*n* = 71)		133.0													5.4				
CMV syndrome	+ (*n* = 6)		41.3													8.3				
	Ø (*n* = 71)		133.0													5.4				
Invasive CMV disease	+ (*n* = 2)		7.0	-16.0													15.0	1.6		
	Ø (*n* = 75)		129.1	1.8													9.3	5.1		
**Patient sex**	Female (*n* = 31)					8.1	55.4					-.3				4.8		4.3	4.3	
	Male (*n* = 46)					6.7	40.1					1.5				6.1		5.5	2.4	
**Immunosuppressvie drugs**																				
mTOR	+ (*n* = 12)							3.4												
	Ø (*n* = 65)							.9												
